# ctDNA as a novel and promising approach for cancer diagnosis: a focus on hepatocellular carcinoma

**DOI:** 10.17179/excli2023-6277

**Published:** 2023-08-03

**Authors:** Negin Biglari, Mohammad Sadegh Soltani-Zangbar, Jamal Mohammadian, Amir Mehdizadeh, Khadijeh Abbasi

**Affiliations:** 1 Department of Animal Biology, Faculty of Natural Sciences, University of Tabriz, Tabriz, Iran; 2 Connective Tissue Diseases Research Center, Tabriz University of Medical Sciences, Tabriz, Iran; 3 Department of Immunology, Faculty of Medicine, Tabriz University of Medical Sciences, Tabriz, Iran; 4 School of Advanced Medical Science, Tabriz University of Medical Sciences, Tabriz, Iran; 5 Hematology and Oncology Research Center, Tabriz University of Medical Sciences, Tabriz, Iran; 6 Department of Biochemistry and Clinical Laboratories, Faculty of Medicine, Tabriz University of Medical Sciences, Tabriz, Iran

**Keywords:** ctDNA, hepatocellular carcinoma, cancer diagnosis, screening biomarkers, ctDNA detection

## Abstract

Hepatocellular carcinoma (HCC) is one of the most prevalent forms of cancer worldwide. Therefore, it is essential to diagnose and treat HCC patients promptly. As a novel discovery, circulating tumor DNA (ctDNA) can be used to analyze the tumor type and the cancer location. Additionally, ctDNA assists the cancer stage determination, which enables medical professionals to provide patients with the most appropriate treatment. This review will discuss the HCC-related mutated genes diagnosed by ctDNA. In addition, we will introduce the different and the most appropriate ctDNA diagnosis approaches based on the facilities.

## List of abbreviations

AFP alpha-fetoprotein

AFP-L3 Lens culinaris agglutinin-reactive AFP

ASH alcoholic steatohepatitis

AUC area under the ROC curve

BEAMing "Beads, emulsion, amplification, and magnetics"

CAPP-seq cancer personalized profiling by deep sequencing

cfDNA cell-free DNA

CTC Circulating tumor cells

ctDNA Circulating tumor DNA

CTNNB1: catenin beta 1

DCP Des-carboxyprothrombin

ddPCR droplet digital polymerase chain reaction

DFS disease-free survival

E2F1 E2 promoter binding factor 1

ECM extracellular matrix

EDTA ethylene diamine tetraacetic acid

EPCAM epithelial cellular adhesion molecule

FISH fluorescent in situ hybridization

FNA fine needle aspiration

GP73 Golgi Protein-73

GPC3 Glypican-3

HANM HCC-associated NAFLD gene module

HBV Hepatitis B virus

HCC Hepatocellular carcinoma

HCV Hepatitis C virus

LEN Lenvatinib

MDCT: multi-detector helical CT

MRI Magnetic resonance imaging

MAFLD metabolic-associated fatty liver disease

NASH nonalcoholic steatohepatitis

NCOR1 nuclear receptor corepressor 1

NGS Next-generation sequencing

PDGFRA platelet derived growth factor receptor alpha

PIK3CA "Phosphatidylinositol-4,5-Bisphosphate 3-Kinase Catalytic Subunit Alpha"

RUNX3 Runt-associated transcription factor 3

SNVs single-nucleotide variation

Tam-Seq Tagged-amplicon deep sequencing

TCF1 T cell factor 1

TERT Telomerase reverse transcriptase

TGF-β Tumor growth factor beta

TP53 tumor protein 53

WES whole exome sequencing 

WGBS-Seq whole genome bisulfite sequencing

WGS whole genome sequencing

YY1 Yin Yang 1

ZNF143 Zinc Finger Protein 143 

## Introduction

Hepatocellular carcinoma (HCC) is considered the seventh most common cancer among women and the fifth among men (Li et al., 2018[93]; Ren et al., 2013[129]) and is known as a progressive malignancy with a poor prognosis; therefore, early diagnosis plays a vital role in the treatment and multiple curative therapeutic options (Yang et al., 2022[166]). About 2-4 % of patients with cirrhosis (infectious, metabolic, or alcoholic etiology) and HBV are high-risk factors for HCC (Daoudaki and Fouzas, 2014[31]). Therefore, clinical practice guidelines for high-risk patients, such as ultrasound imaging with or without serum alpha-fetoprotein (AFP) testing, are recommended each two years (Galle et al., 2018[51]). Studies show a low sensitivity level of the ultrasound method in patients with cirrhosis. Hence, combining two ultrasound and AFP methods can considerably increase the sensitivity of HCC detection in clinical practice; however, these methods are also insufficient (Tzartzeva et al., 2018[157]).

cfDNA is a biological marker as a circulating cell-free DNA. cfDNA is a fragmented blood circulation molecule detected in healthy individuals and patients with or without cancer (Kaseb et al., 2019[81]). Another form of cfDNA is ctDNA, which is derived from apoptotic or necrotic tumor cells or produced from phagocytized necrotic tumor cells by macrophages (Alix-Panabières et al., 2012[3]). Segregation of ctDNA from cfDNA is performed based on cancer genetic or epigenetic changes. Therefore, this kind of DNA would be considered a novel diagnosis biomarker (Yang et al., 2019[167]).

**Figure 1:**
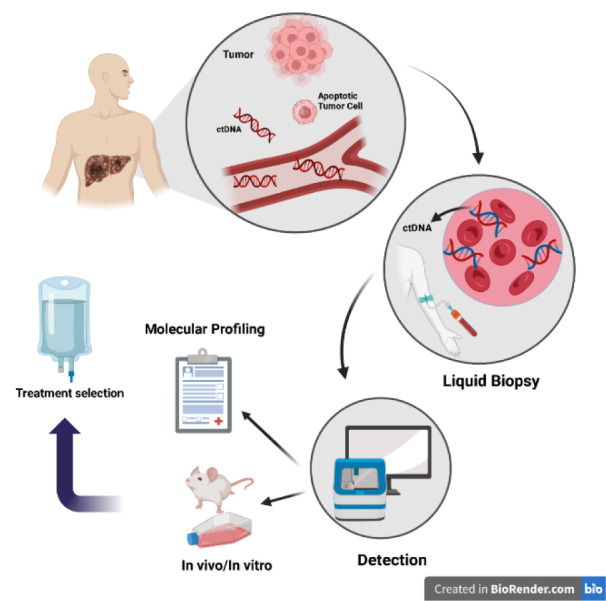
ctDNA can be used as a biomarker to identify hepatocellular cancer. Cancerous DNA escapes from blood cells at the beginning of the process and spreads throughout the body. In the second stage, ctDNA can identify the condition by taking a blood sample from the patient. In the third stage, detecting the mutant gene enables physicians to determine which state the tumor is in; based on this information, they can choose the most appropriate treatment for the patient. Created with BioRender.com

In recent years, examining plasma blood cells and ctDNA analysis has provided a non-invasive method to detect HCC in its early stages. This review will discuss the fundamentals of HCC diagnosis and therapy, ctDNA and involved genes, ctDNA detection methods, and their limitations. Figure 1(Fig. 1) briefly shows the application of ctDNA as a biomarker is shown.

## HCC: Physiopathology and Epidemiology

Due to different risk factors, such as carcinogens and viral hepatitis, the incidence of HCC shows a substantial global variation (El-Serag, 2012[42]). Studies have indicated two significant factors, HBV and HCV, playing a prominent role in disease formation (Tarocchi et al., 2014[150]). In this regard, viral DNA integration in the host genome can lead to HCC induction by HBV. At this stage, liver cells enter the initial phase of natural acute infections. Following HBV-DNA insertion in the human genome, genetic alterations such as gene and chromosomal deletion and translocations, generalized genomic instability, amplification of cellular DNA, and the generation of fusion transcripts will occur in cells (Feitelson and Lee, 2007[45]). In this regard, the oncogenes expression and tumor suppressor genes can be affected by this integration. Several mechanisms, such as immune defense control systems, viral inhibition of antigen presentation, selective immune suppression, down-regulation of viral genes, and viral mutations, may lead to HCC progression, which causes functionally disabled virus-specific T cells to identify HBV antigen (Ortega-Prieto and Dorner, 2017[118]).

### Epidemiology

Globally, HCC is the most frequent type of liver cancer. In this regard, the HCC rate among men is higher than that of women (13.6 versus 4.7 per 100.000 persons, respectively), and this rate is mainly dependent on age, gender/sex, and race (McGlynn et al., 2021[106]). The incidence of HCC is also significantly correlated with age. However, in the other groups, the typical age of patients varies at the time of diagnosis. For example, in South Korea in 2018, the average age of diagnosed HCC patients was 60-79 (Chon et al., 2021[26]). In China and Taiwan, the median age of diagnosis is 63 years, and 16.1 % of diagnosed patients are older than 75 (Honda et al., 2011[68]).

In the studies conducted by Japanese researchers in 2011, among the 624 studied HCC patients, 478 were men, and 146 were women (Honda et al., 2011[68]). Additionally, in another study in the United States, approximately 78 % of patients were men (Kennedy et al., 2018[84]). However, in some countries, such as Uganda and Costa Rica, the infection rate of men and women is almost equal (Petrick et al., 2020[122]). In the United States, 67 % of HCC patients are 60 or older (Petrick et al., 2020[122]).

The difference in disease prevalence is more evident in countries with high racial diversity, such as the United States. For instance, in the United States, HCC rates are two times higher in Asians than in African Americans, whose rates are two times higher than in whites. The reason probably is due to the differences in the prevalence and gaining time of significant risk factors for liver disease and HCC (El-Serag and Rudolph, 2007[43]). Japanese Americans (n = 142; 40 %) were the main racial/ethnic group identified with HCC in a study done in 2021 on 359 patients in the United States. Latinos (n = 106; 29 %), whites (n = 46; 13 %), African Americans (n = 42; 12 %), and Native Hawaiians (n = 23; 6 %) were the next most prevalent racial/ethnic groups (Barzi et al., 2021[9]).

### Physiopathology

The tissue environment is one of the most crucial elements which profoundly influences tumor formation and growth (Yang et al., 2011[168]). A typical cell transitioning into a preneoplastic lesion and, finally, a malignant tumor is known as carcinogenesis (Severi et al., 2010[135]). Direct or indirect interaction of different cell types in the tumor stroma with components of the extracellular matrix (ECM), such as collagen, fibronectin, laminin, glycosaminoglycans, hyaluronan, and proteoglycans, can lead to unusual phenotype formation, promoting this transformation (Schrader and Iredale, 2011[133]). Depending on the mass of the tumor and the presence of liver cirrhosis, the tumor's appearance can be different. In this regard, different types of HCC include single mass, multi-nodular with many tumors scattered throughout the liver, or a diffusely growing lesion. Multicentric HCCs are usually observed in patients with chronic hepatitis C. These tumors are generally multi-nodular HCCs (Röcken and Carl-McGrath, 2001[131]).

Except for rare fibrolamellar varients tumors, most HCC tumors are soft. Depending on the lipid changes or bile production, the color of the cut surface of these tumors varies from yellow or green to, in some cases, tan-brown or gray-white. Although, in some cases, there is an invasion of the hepatic vein, bile ducts, and portal vein thrombosis to HCC tumors (Röcken and Carl-McGrath, 2001[131]).

Known as a heterogeneous disease, metabolic-associated fatty liver disease (MAFLD) is one of the essential risk factors for HCC (Burt et al., 2015[18]). In one study, coexpressed gene modules were extracted for MAFLD and HCC (Ge et al., 2021[55]). In MAFLD, four coexpressed gene modules were identified. The HCC-associated MAFLD gene module (HANM) is significantly overlapped with HCC. In this regard, YY1, E2F1, and ZNF143, known as transcription factors, had significant overexpression in HANM, showing the survival importance in HCC patients (Ge et al., 2021[55]). As nonalcoholic steatohepatitis progresses, it can result in HCC (Yasui et al., 2011[170]). Hepatic steatosis can also lead to inflammation, fibrogenesis, and oxidative damage to liver cells (Huang et al., 2021[72]). The down-regulation of Runt-associated transcription factor 3 (RUNX3) is directly associated with NASH; however, this gene is absent in patients with alcoholic steatohepatitis (ASH) (Sookoian et al., 2020[140]).

In a study conducted on 371 patients, three common HCC molecular subclasses were demonstrated. The subclass mapping method of this study indicated three robust subclasses named S1, S2, and S3. In this research, subclass S2 was more considerable than others, while the S3 subclass was minor and included well-differentiated tumors. However, S3 was better diagnosed, whereas S1 and S2 had poor diagnoses (Hoshida et al., 2009[69]).

Some molecular pathways such as TP53, WNT/catenin, (Tumor growth factor-β) TGF-β, TERT Telomerase reverse transcriptase, etc., also have a prominent role in HCC formation (Wu et al., 2020[164]).

Additionally, the TGF-β signaling pathway plays an essential role in HCC progression. During carcinogenesis, TGF-β plays a dual function on the malignant cell, behaving as a suppressor factor at the early stages. However, once cells escape its cytostatic effects, this pathway contributes to later tumor progression (Gonzalez-Sanchez et al., 2021[60]). Generally, in S1 and S3 subtypes, TGF-β and WNT/β-catenin pathways are usually activated. WNT/β-catenin pathway also plays a prominent role in HCC pathogenesis. Conversely, the S2 subtype is characterized by high AFP, EPCAM, and GPC3 expression, as the main hepatic stem cell markers (Ulz et al., 2016[158]; Yamashita et al., 2009[165]).

By identifying 16 mutated genes in HCC tumors, researchers classified the HCC tumors into six subgroups, including G1 to G6.

G1-G3 subgroups show a high chromosomal instability; these subgroups also have PIK3CA mutated genes. However, the G1 subgroup is associated with HBV infection with low viral DNA copies, AXIN1 mutation, and high serum AFP levels. The G2 subgroup is also associated with HBV infection and high viral copies. This subgroup also shows frequent local and vascular invasion with TP53 mutation. Despite the two last subgroups, G3 didn't indicate HBV infection; mainly, these tumors included TP53 mutation.

**Figure 2:**
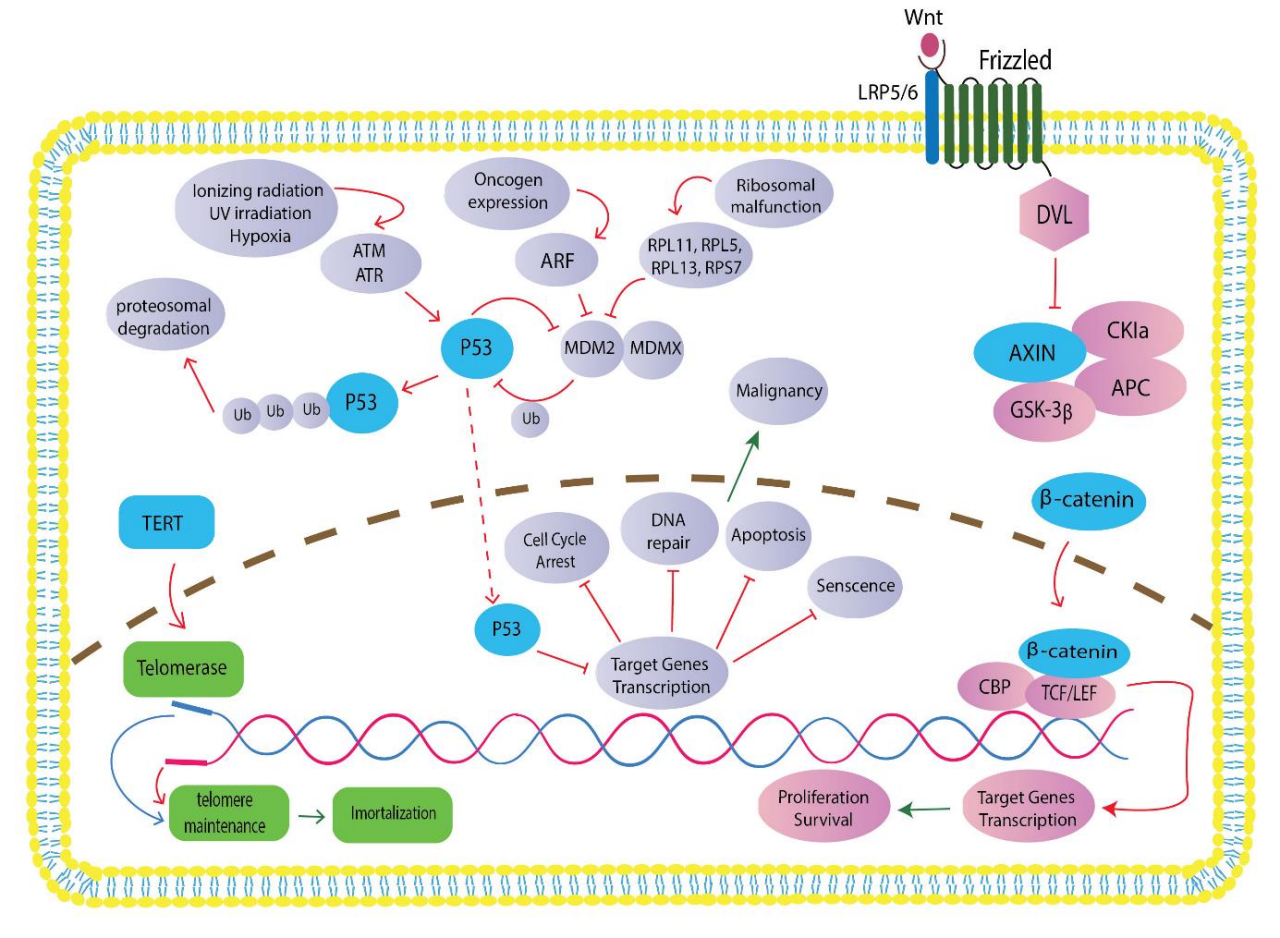
TERT signaling upon mutation (marked with green), p53 signaling pathway (marked with blue), and Wnt-catenin pathway (marked with red) are the typical signaling pathways in HCC development. They influence carcinogenesis and development of hepatocellular carcinoma by interacting with some common oncogenes and tumor suppressors. These tasks involved: proliferation, immortalization, genomic stability, cell differentiation, and survival. The ctDNA mutation genes play essential roles in these pathways.

The G4 subgroup included non-tumorous liver tissue, which clustered tightly together. These tumors mostly have a TCF1 mutation. β-catenin activation is also highly related to G5 and G6 tumors, so 70 % to 100 % of these tumors contained CTNNB1 mutation (Boyault et al., 2007[15]; Yim and Lee, 2021[173]). Figure 2(Fig. 2) summarizes the involved molecular pathways in HCC.

## HCC Diagnosis and Management

Given that most HCC cases can be identified, namely those with cirrhosis or chronic hepatitis B, many patients are discovered by monitoring (Papatheodoridis et al., 2016[120]; Singal et al., 2020[137]). HCC screening is recommended in high-risk populations to reduce the mortality rate potential and increase the treatment efficacy (Bruix et al., 2014[17]). Since less than 20 % of individuals with cirrhosis undergo HCC screening, most instances are found at an advanced stage. How soon an individual should get tested for HCC depends on their risk and whether or not they plan to undergo treatment if they are diagnosed with the disease (Dalton-Fitzgerald et al., 2015[30]; Davila et al., 2011[32]). According to the guidelines of many scientific bodies, HCC screening should be undertaken every six months, as this frequency results in better survival rates than annual surveillance and comparable outcomes to those achieved within a three-month interval (Trinchet et al., 2011[156]). However, there are still some discrepancies over the best monitoring methods. New evidence shows that abdominal ultrasonography may give poor diagnostic data in patients, for example, with obesity and nonalcoholic steatohepatitis (NASH) (Atiq et al., 2017[6]).

### Ultrasound

The best screening method is liver ultrasound, which may be done twice a year. This method is non-invasive, widely accessible, and cost-effective. It also has a sensitivity of 60 % to 80 % and a specificity of more than 90 % (Tejeda-Maldonado et al., 2015[153]). Recently, sonographic contrast agents such as intra-arterial carbon dioxide and helium have potentially increased the accuracy of this method. Meanwhile, the evaluation of intrahepatic vascular flow can significantly benefit from duplex and color Doppler sonography. Compared to metastatic or hemangiomas, HCC lesions often exhibit delicate branching patterns of increased vascularity and higher flow rates (Choi et al., 2002[25]; Nishiharu et al., 1998[112]). Technological advancements and ultrasound technicians' skills contribute to the technique's sensitivity. As a result, highly skilled technicians and physicians must carry out ultra-sonographic screening in dedicated facilities (Galle et al., 2018[51]). The reduced sensitivity of ultrasound in patients with obesity and non-viral liver illness is one of the limitations of this diagnostic method. Liver nodularity and the patient's capacity to quickly stop breathing are two other examples of patient characteristics that can affect ultrasound diagnostic accuracy (Del Poggio et al., 2014[33]).

### Alpha-fetoprotein (AFP)

The most well-studied blood biomarker for HCC is AFP. Other biomarkers, such as Lens culinaris agglutinin-reactive AFP (AFP-L3), Des-carboxyprothrombin (DCP), Golgi Protein-73 (GP73), and Glypican 3 (GPC3), are currently being investigated and need more validations (Rich and Singal, 2014[130]). However, in one study, the diagnostic performance of AFP was unsatisfactory, so the sensitivity and specificity of the 20 ng/mL AFP detection threshold for HCC ranged from 41 %-65 % and 80 %-94 %, respectively (Huang et al., 2016[71]). The most popular cut-off used in clinical practice is 20 ng/mL; however, this level was obtained from a study in which only one-third of the patients had early HCC (Trevisani et al., 2001[155]). Due to the positive relationship between AFP levels and tumor burden, cut-off lowering can increase the sensitivity for early-stage detection. Using a lower cut-off of 10.9 ng/mL, AFP demonstrated a sensitivity as high as 66 % for early-stage HCC in a multicenter phase II biomarker trial on 836 patients (419 with HCC and 417 with cirrhosis) (Marrero et al., 2009[104]).

### Screening biomarkers

Discovering sufficiently robust serological markers for identifying HCC remains essential in clinical practice. Current liquid biopsy research has yielded encouraging results in finding novel screening techniques for this condition. The so-called liquid biopsy is a minimally invasive process identifying tumor components secreted into biological fluids, most notably blood samples. Circulating tumor cells (CTCs), circulating tumor nucleic acids, DNA (ctDNA) and RNA (miRNA and long non-coding RNA), and extracellular vesicles are among the tumor components (Mann et al., 2018[103]).

### Other methods

Additional to the ultrasound, other techniques, such as endoscopy, are employed to identify HCC. Endoscopy and fine needle aspiration (FNA) can improve the HCC staging compared to CT and MRI (Jasirwan et al., 2020[78]). Early arterial phase images (18-28 s after contrast injection), as well as late (early parenchymal) arterial phase images, can also be retrieved using multi-detector helical CT (MDCT).

The vessels in individuals being considered for surgical resection are clarified more optimally in the latter phase. However, lesions are better demonstrated in the "late" arterial phase than in the "early" arterial phase (Baron and Brancatelli, 2004[8]).

MRI is a different technique for HCC diagnosis. Obtaining high-resolution pictures of the liver without ionizing radiation or nephrotoxic contrast chemicals is a benefit of MRI over CT. In the early detection and diagnosis of HCC, MRI is just as accurate as helical CT (Szklaruk et al., 2003[146]).

## Circulating Tumor DNA (ctDNA)

Cell-free DNA (cfDNA) and ctDNA are two kinds of circulating DNA (approximately 167bp) (Ulz et al., 2016[158]), released from nonmalignant and malignant cells, respectively. The difference between these two DNA molecules is mainly related to their origin. In 1984 Mandel and Metais (1948[102]) described cfDNA as a circulating cell-free DNA broken into small DNA molecules and released into the bloodstream. Extracted by centrifugation in EDTA tubes (Lo et al., 1999[97]; Yang et al., 2019[167]), its detection is complicated, and specific methods are needed.

It is thought by active and passive processes, both normal and tumor cells release cfDNA into the bloodstream. ctDNA is derived from apoptotic and necrotic tumor cells (Mouliere et al., 2011[107]). In principle, by diagnosis and investigation of ctDNA, genetic defects of the tumor cells can be detected regarding their cell of origin. It can be said that ctDNA is a small part of the whole cfDNA in the bloodstream. By identifying and tracking tumor-specific mutations in circulating cfDNA, the presence of ctDNA can also be detected (Crowley et al., 2013[29]). The amount of circulating tumor-derived DNA varies between 0.01 % and 50 % (Bettegowda et al., 2014[12]). However, accurate and efficient ctDNA detection techniques for tumor diagnosis are essential. A study conducted on 72 patients with HCC and 37 healthy individuals observed a significantly higher level of cfDNA in affected individuals than in controls and individuals with benign tumors. The cfDNA levels were also absolutely correlated with tumor size. The area under the ROC curve (AUC) value for distinguishing HCC from the HC group (healthy controls groups) and benign patients was 0.949 and 0.874, respectively (Huang et al., 2012[73]).

## CtDNA Fundamentals

ctDNA is an extracellular DNA originating from apoptotic or necrotic cancer cells. This molecule can be found in physiological fluids such as blood, synovial, and cerebrospinal fluid and comprises single or double-stranded DNA (Li et al., 2019[92]). Because of its non-invasive and repeatable evaluation, ctDNA has steadily been developed from research to clinical application, making it a promising tool for discovering gene alterations. Although the existence of ctDNA is generally established, the processes by which tumor DNA reaches circulation remain unknown (Zhao et al., 2018[178]).

It has been proposed that there are three possible sources for ctDNA: I) apoptotic or necrotic tumor cells, II) live tumor cells, and III) circulating tumor cells (Cheng et al., 2016[24]). In this regard, changes in ctDNA level correctly indicate the concurrent disease state of six immunotherapy cases across the course of the disease. In contrast, a rise in ctDNA level following therapy accurately predicts the disease progression (Gray et al., 2015[62]). Studies have also shown increased ctDNA levels before radiological evidence indicating the disease progression and acquired resistance to targeted therapy. ctDNA quantification has also recently been highlighted as a suitable complementary modality to functional imaging for real-time monitoring of tumor burden (Gray et al., 2015[62]; Wong et al., 2017[163]). Therefore, because there is a strong association between the disease state and plasma DNA, ctDNA can possibly be used in clinical diagnosis in addition to imaging scans to help HCC tumors dynamics monitoring.

## CtDNA and Mutations

Different studies have indicated that molecular alterations in HCC ctDNA mainly include copy number variation, DNA methylation aberration, hepatitis B virus (HBV) integration, and single-nucleotide variation (SNVs) (Hlady et al., 2019[67]).

Eight investigations on various populations have established the ability to use the TP53 gene mutation as a ctDNA identification marker. 

In a study conducted by An et al. (2019[4]) on 26 HCC patients' ctDNA, out of 139 somatic mutations and 93 genes, the TP53 gene was mutated in 50 % of the patients. Moreover, 11.54 % of patients had mutations in AXIN1, BCL6 Corepressor (BCOR), catenin beta 1 (CTNNB1), Fanconi Anemia Complementation Group E (FANCE), Fanconi anemia, complementation group M (FANCM), and nuclear receptor corepressor 1 (NCOR1) genes (An et al., 2019[4]).

In another study by Jeppesen et al. (2019[79]) on 28 HCC patients, mutations in 19 genes were observed in tumor tissues and plasma of 21 patients. Hence, the TP53 gene included 33 % of the gene mutations, indicating the importance of this gene in diagnosing the disease stages. PDGFRA and KIT gene mutations were also observed in 24 % and 19 % of the patients, respectively (Jeppesen et al., 2019[79]).

A study on patients with advanced HCC in Japan also demonstrated that somatic alterations could be distinguished in most advanced HCC patients by ctDNA profiling and ctDNA-kinetics during lenvatinib (LEN) treatment as a valuable marker for disease progression monitoring. In this study, in a total of 131 single nucleotides analysis, 54 %, 42 %, 25 %, and 13 % of patients showed a mutation in tumor protein 53 (TP53), CTNNB1, Ataxia telangiectasia mutated (ATM), and AT-Rich Interaction Domain 1A (ARID1A), respectively (Fujii et al., 2021[49]).

The CTNNB1 gene has also been used as another marker in HCC investigations. In a study. The CTNNB1 mutation (CTTNB1P.T41A) rate was 9.5 % (9/95) in 95 cancer patients' plasma samples in the pre-treatment samples; however, when plasma analysis results were added to the subset of patients with accessible tissue samples, the mutation detection rate rose to 13.5 % (5/37) (Oversoe et al., 2021[119]).

In several studies, the telomerase reverse transcriptase (TERT) gene mutations have also been investigated in ctDNA samples. In one study, 124 bp G>A hTERT promoter mutation was observed in 36 HCC patients' serum samples (69 %), and no significant differences were found between the traits of the serum mutation-positive and serum mutation-negative patient groups. However, the patients' disease-free survival (DFS) with the mentioned mutation was considerably shorter than that of the serum mutation-negative patients (Ako et al., 2020[2]).

ctDNA mutation analysis of 26 advanced HCC patients also showed that TERT C228T and TP53 mutations were at higher levels, so 77 % of the patients showed C228T mutation in the TERT promoter (Ge et al., 2021[56]).

Moreover, the most frequently mutated genes in Atezo/Bev-treated u-HCC patients were TP53 and CTNNB1 detected in the TERT promoter in ctDNA. Therefore, in u-HCC patients receiving Atezo/Bev, cfDNA/ ctDNA profiles can help the therapy results outcome (Matsumae et al., 2022[105]).

TERT, CTNNB1, and TP53 have been investigated in two separate studies and different populations. ddPCR can detect these mutations to analyze intratumoral heterogeneity. Hence, ctDNA detection may be a promising liquid biopsy in HCC management (Huang et al., 2016[71]; Liao et al., 2016[96]).

## TP53

The gene TP53 is on the short arm of human chromosome 17 (17p13.1) and comprises 11 exons, ten introns, and 393 amino acid residues. The p53 protein is a transcription factor typically categorized into three functional domains: the amino-terminal domain, the DNA binding domain, and the carboxy-terminal domain (Borrero and El-Deiry, 2021[14]). TP53 is the most commonly altered gene in human cancers and functions as a multifunctional transcription factor that regulates DNA replication and repair, genomic stability maintenance, cell cycle progression, and programmed cell death. By integrating internal and external stimuli, p53 determines the cell fate of compromised cells based on the extent of cell damage and the feasibility of repairing impaired cell structures. Therefore, p53 acts as a genuine cellular controller and tumor suppressor gene that prevents the abnormal proliferation of transformed cells (Pollutri et al., 2016[125]).

In comparison to other suppressors of tumors, p53 displays a unique attribute in which mutations can produce diverse impacts on its operation, leading to outcomes such as loss-of-function (LOF), dominant-negative (DN), and gain-of-function (GOF) (Kazanets et al., 2016[83]). Moreover, various research studies have provided evidence that particular mutations in the p53 gene result in gain-of-function characteristics, thereby causing p53 to act as an oncogene rather than a suppressor of tumors. Specific mutations in the p53 gene have also been observed to facilitate the capacity of cancerous cells to sustain proliferation, intensify their aggressiveness, encourage metastasis, and induce resistance to chemotherapy (Borrero and El-Deiry, 2021[14]).

Additionally, the transcription factors p73 and p63 are encoded by the genes TP73 and TP63, respectively, on chromosome 1p36.33 and chromosome 3q28-the transcription factors in question display structural homology with TP53 (Müller et al., 2006[108]). The domain responsible for binding to DNA exhibits a remarkable level of protection across the various members of the p53 family, with the oligomerization domain displaying a similar level of conservation in proximity. Likewise, it can be observed that the transcriptionally active (TA) domain shows the least amount of protection. The resemblances noted between p73 and p63 facilitate their ability to participate in oligomerization, attach to typical p53 response elements (REs), and stimulate the transcription of p53 target genes (Stiewe et al., 2004[142]; Tannapfel et al., 2008[149]).

Previous research has indicated the importance of p63 and p73, which belong to the p53 gene family, not only in embryonic development and differentiation but also in the induction of programmed cell death and the reaction of hepatocellular carcinoma for therapeutic interventions. Activating the p53 family is critical in the DNA damage response, chemosensitivity, and prognosis of hepatocellular carcinoma. 

The p53 family consists of TP53, TP63, and TP73 and plays a role in the tumor suppressor and oncogenic capacities of p53. Numerous isoforms of TP63 and TP73 can be attributed to the alternative utilization of promoters for transcription and splicing. Isoforms of p63 and p73 are extended in length and contain a transactivation domain (TAD), which can activate the same target genes as p53 and trigger apoptosis. Conversely, shortened p63 or p73 isoforms at the amino terminus function as dominant negatives with opposing effects through DN mechanisms (Ghioni et al., 2002[57]; Yoon et al., 2015[175]).

The influence of the HCC on the apoptotic signaling pathway facilitated by the p53 family appears in varying degrees of diversity. Therapy resistance and poor prognosis in patients with HCC are frequently associated with p53 mutation and an imbalanced ratio of TA and DN-isoforms of p63 and p73 (Kunst et al., 2016[91]). DNp73 has been observed to interfere with apoptosis and chemosensitivity at various stages of the apoptosis signaling pathway. The transcription of CD95, TNF-R1, TRAIL-R2, and TNFRSF18, which are responsible for encoding death receptors, is subjected to negative regulation by DNp73.

Furthermore, DNp73 demonstrates repressive effects on the genes that encode caspase-2, -3, -6, -8, and -9. Concurrently, DNp73β exerts a repressive effect on apoptosis induced by mitochondrial dysfunction. The adverse selection of the TA isoforms' death receptor and mitochondrial apoptosis activity has also been observed in the expression of DNp73 in HCC (Schuster et al., 2010[134]).

The fundamental mechanism underlying the GOF activity of mutant p53 in HCC is attributed to its capacity to bind and disable the TA isoforms of p63 and p73. The impact of p53 GOF mutants on regulating pro-apoptotic genes that govern the extrinsic and intrinsic apoptosis signaling pathways in HCC results in compromised apoptosis and drug resistance. Hence, directing attention toward the interplay between GOF mutant p53 proteins and TAp63 and TAp73 appears viable for prospective HCC treatment (Oren and Rotter, 2010[117]; Soussi and Wiman, 2015[141]).

## TERT

The TERT gene is located on chromosome 5p15.33 in humans and plays a crucial role as a constituent component of the telomerase holoenzyme. The TERT gene spans a length of 42 Kb and is composed of 15 introns and 16 exons. Its promoter core also measures 260 base pairs (Akincilar et al., 2016[1]).

The reverse transcriptase domain is encoded by transcribing 5-9 exons. It has been observed that the TERT transcript can undergo splicing, producing 22 isoforms (Takakura et al., 1999[147]). The TERT promoter (TERTp) region comprises GC boxes, which have an affinity for the zinc finger transcription factor Sp1, thereby increasing TERT transcription. Additionally, E-boxes in this region can bind transcriptional enhancers and repressors. However, the TERTp region does not possess a TATA box, harboring multiple binding sites for various transcription factors (Hrdličková et al., 2012[70]).

The process of carcinogenesis in human cancers, including HCC, is often characterized by telomere shortening and telomerase reactivation. The telomere shortening and subsequent telomerase reactivation have been identified as a genetic predisposition for developing liver cirrhosis and liver cancer (Kumar et al., 2016[90]). TERT promoter mutations represent an instance of this phenomenon. Telomerase activation is essential in developing liver cancer, as documented in more than 80 % of HCCs. Telomerase reactivation has been linked to increased TERT and TERC expression (in der Stroth et al., 2020[75]). TERT and telomerase activation or re-expression occur early in the premalignant progression of cirrhotic liver and regenerative nodules. Mutations in telomerase have been associated with various human diseases, including congenital dyskeratosis, aplastic anemia, familial idiopathic fibrosis, and acute myeloid leukemia (Kirwan et al., 2009[86]). The presence of mutations leads to impaired tissue regeneration, which can be attributed to the dysfunction of telomeres and the loss of stem/progenitor cells. The genetic alterations observed in the liver cancer samples were analogous. The study additionally reveals the existence of TERT and TERC mutations among individuals who have been clinically diagnosed with hepatic cirrhosis. The study conducted a comparative analysis between the HCC patients and a control group of healthy individuals, utilizing buccal mucosa tissue and peripheral blood as the primary biological samples. Cohort studies investigating hepatic cirrhosis have demonstrated a higher prevalence of telomerase mutations (Cillo et al., 2016[28]). Calado et al. (2011[19]) identified the genetic mutations in the TERT gene in nine individuals and the TERC gene in one individual in a cohort study among 134 patients diagnosed with liver cirrhosis. Mutations in TERT and TERC genes were also detected by Hartmann et al. (2011[64]) in 16 out of 521 patients. According to the Calado study, there was a greater incidence of gene variants in the TERT and TERC genes among individuals with cirrhosis compared to the control group. According to the Hartmann study, telomerase mutations are higher in cirrhosis individuals than in healthy controls.

The research results on TERT alterations also suggest that TERT promoter mutations may be a more effective predictor of patient outcomes in early-stage HCC cases that have undergone surgery. Conversely, TERT expression may be more closely linked to the prognosis of advanced-stage HCC patients eligible for non-surgical treatments. In contrast, a correlation has been reported between the length of telomeres and survival after hepatectomy. This aligns with the results of various studies suggesting the possible prognostic importance of telomere length in a subset of patients diagnosed with HCC (Jang et al., 2021[77]).

## CTNNB1/AXIN1

The CTNNB1 gene is a crucial oncogene that plays a significant role in developing HCC. The CTNNB1 gene encodes the β-catenin protein, which is critical in several cellular processes. The β-catenin protein facilitates intercellular adhesion and communication within adherent junctions (Khalaf et al., 2018[85]). The development of HCC is frequently linked to genetic abnormalities, with exon 3 deletions or missense mutations as the most prevalent abnormalities. Missense mutations in CTNNB1 are associated with elevated expression of β-catenin, stimulating the Wnt pathway and playing a role in the pathogenesis of HCC (Kondo et al., 1999[89]; Lu et al., 2014[99]; Vilchez et al., 2016[160]). A study assessed the correlation between β-catenin mutation and HCC progression. The findings of this investigation indicate a favorable correlation between the β-catenin mutation and the manifestation of high-grade differentiation in HCC (Kitao et al., 2015[87]).

Moreover, the mutation of β-catenin substantially amplifies pseudo-glandular proliferation and bile synthesis in HCC. The HCC development is also facilitated by increased production of truncated β-catenin proteins, attributed to abnormal localization and β-catenin mutation. Both conventional and missense mutations have the ability to impact the expression of β-catenin in HCC and facilitate its progression (Li et al., 2014[94]). According to reports, a subset of HCC patients (about 12.8 %) exhibit conventional mutations in codons 33, 37, 41, and 45 (Okabe et al., 2016[115]). Missense mutations may also arise within codons 32, 34, and 35. Hence, mutations such as conventional and missense in the CTNBB1 gene can augment the expression of β-catenin and instigate its translocation to the nucleus (Deldar Abad Paskeh et al., 2021[34]).

The activation of the Wnt pathway and subsequent accumulation of β-catenin resulting from CTNNB1 mutation has also been linked to the development of small, well-to-moderately differentiated tumors and a favorable prognosis (Khalaf et al., 2018[85]). Mutations in β-catenin in HCC are associated with increased incidences of microvascular and macro-vascular invasion, larger tumor size, and the development of multiple nodules (Tien et al., 2005[154]). The impact of β-catenin mutations on human HCC depends on whether they are GOF or inactivating mutations of AXIN1. HCCs linked to hepatitis B virus (HBV) infection are also characterized by Axin and rare β-catenin mutations. Conversely, HCCs not associated with HBV infection are primarily marked by β-catenin mutations and exhibit a well-differentiated and chromosomally stable phenotype (Zucman-Rossi et al., 2007[179]).

Moreover, the mutations in the destruction complex contribute to HCC development. Mutations in AXIN1 and AXIN2 have been observed in a considerable proportion of HCC instances, ranging from 2.7 % to 54.2 % (Feng et al., 2012[46]). Mice with AXIN1 mutations develop liver tumors. The β-catenin protein is kept at a low concentration in the cytoplasm by a cytoplasmic destruction complex that lacks Wnt ligands. Adenomatous polyposis coli, glycogen synthase kinase-3 beta, casein kinase-1, and the axis inhibition scaffold proteins AXIN1 and AXIN2 are among the complex members responsible for protein degradation, and the binding of Wnt ligands to their respective receptors. Activating Frizzled and co-receptors LRP5/6 results in the recruitment of disheveled proteins to the cell membrane (Nusse and Clevers, 2017[114]). This recruitment process prevents β-catenin degradation by dissociating AXIN and GSK3β from the cytoplasmic destruction complex. Translating β-catenin into the nucleus activates Wnt target genes via TCF-mediated transcriptional regulation. The gene AXIN1 is recognized as a suppressor of tumor growth and plays a pivotal role in inhibiting the Wnt/β-catenin pathway (Song et al., 2014[139]). Mutations in AXIN1 have also been identified in diverse cancer types. Mutations resulting in AXIN1 truncation or nonsense impede its binding ability to β-catenin, thereby interfering with the destruction complex and activating the Wnt/β-catenin pathway (Qiao et al., 2019[126]).

## ctDNA Detection Methods

Technological advances have facilitated ctDNA detection in blood plasma for scientists. These technologies include digital droplet polymerase chain reaction (ddPCR), (BEAMing) Beads, emulsion, amplification, and magnetics, cancer personalized profiling by deep sequencing (CAPP‑Seq), Taggedamplicon deep sequencing (TAm‑Seq), whole genome sequencing (WGS), whole exome sequencing (WES), and whole genome bisulfite sequencing (WGBS‑Seq) (Table 1(Tab. 1); References in Table 1: Ashida et al., 2016[5]; Bahga et al., 2013[7]; Bratman et al., 2015[16]; Campos et al., 2018[20]; Denis et al., 2016[36]; Deyati et al., 2013[37]; Garrett-Bakelman et al., 2015[53]; Gauri and Ahmad, 2020[54]; Gorgannezhad et al., 2018[61]; Guan et al., 2017[63]; Heitzer et al., 2013[66]; Jin et al., 2018[80]; Li et al., 2021[95]; Lyu et al., 2020[100], 2022[101]; Müller et al., 2006[108]; Newman et al., 2014[110], 2016[111]; Rodda et al., 2018[132]; Sumbal et al., 2018[144]; Sun et al., 2011[145]; Zhang et al., 2019[177]).

### Droplet Digital (dd) PCR

Vogelstein and Kinzler successfully increased the sensitivity of PCR by introducing another technique called ddPCR (Vogelstein and Kinzler, 1999[161]). 

Droplet digital PCR (ddPCR) uses sample partitioning and limiting dilution and Poisson distribution-based statistical data processing to accurately, precisely, and reliably quantify nucleic acids. The admixed nucleic acid molecules and PCR solution are partitioned into many droplets. ddPCR quantifies nucleic acids accurately and sensitively (Pinheiro et al., 2012[123]). The randomized partitioning of sample DNA into isolated droplets utilizing micro-fluidic circuits and surfactant chemistry yields 20,000 droplets with either one or no template DNA. Tandem gene duplications cause copy number changes. Restriction enzymes reliably isolate connected template DNA copies. This method encapsulates and quantifies each sequence in a droplet. Fluorescent Taqman probes help identify "positive" droplets during PCR amplification. Poisson statistics calculate the template DNA concentration by measuring the proportion of "positive" droplets (Jamuar et al., 2016[76]).

There are two main differences between PCR and ddPCR: first, before amplification, ddPCR partitions the PCR reaction into thousands of individual reactions, and second, gaining data at the reaction endpoint (Taylor et al., 2017[151]). Droplet digital PCR is also known as the third-generation PCR. This method is increasingly used in liquid biopsy because of its accuracy in measuring mutations, amplifications, and deletions in DNA (Subhash et al., 2022[143]).

In contrast to qPCR, the ddPCR method possesses several advantageous characteristics. First, it enables absolute quantification by utilizing the principles of sample partitioning in uniform droplets and Poisson statistics correction for multiple target molecules per droplet, thereby circumventing issues related to normalization and calibrator. Second, it has enhanced precision, robustness, and sensitivity in detecting low-target copies. Third, it is relatively impervious to potential PCR inhibitors. Fourth, it measures the absolute number of DNA copies per microliter of reaction, with confidence intervals, a wide dynamic range, and high throughput. Fifth, it is user-friendly, similar to a conventional RT-qPCR.Finally, it can exhibit superior diagnostic performance compared to traditional RT-qPCR (Pinheiro et al., 2012[123]).

The current ddPCR platforms require the implementation of automation and cost-reduction measures.

**Figure 3:**
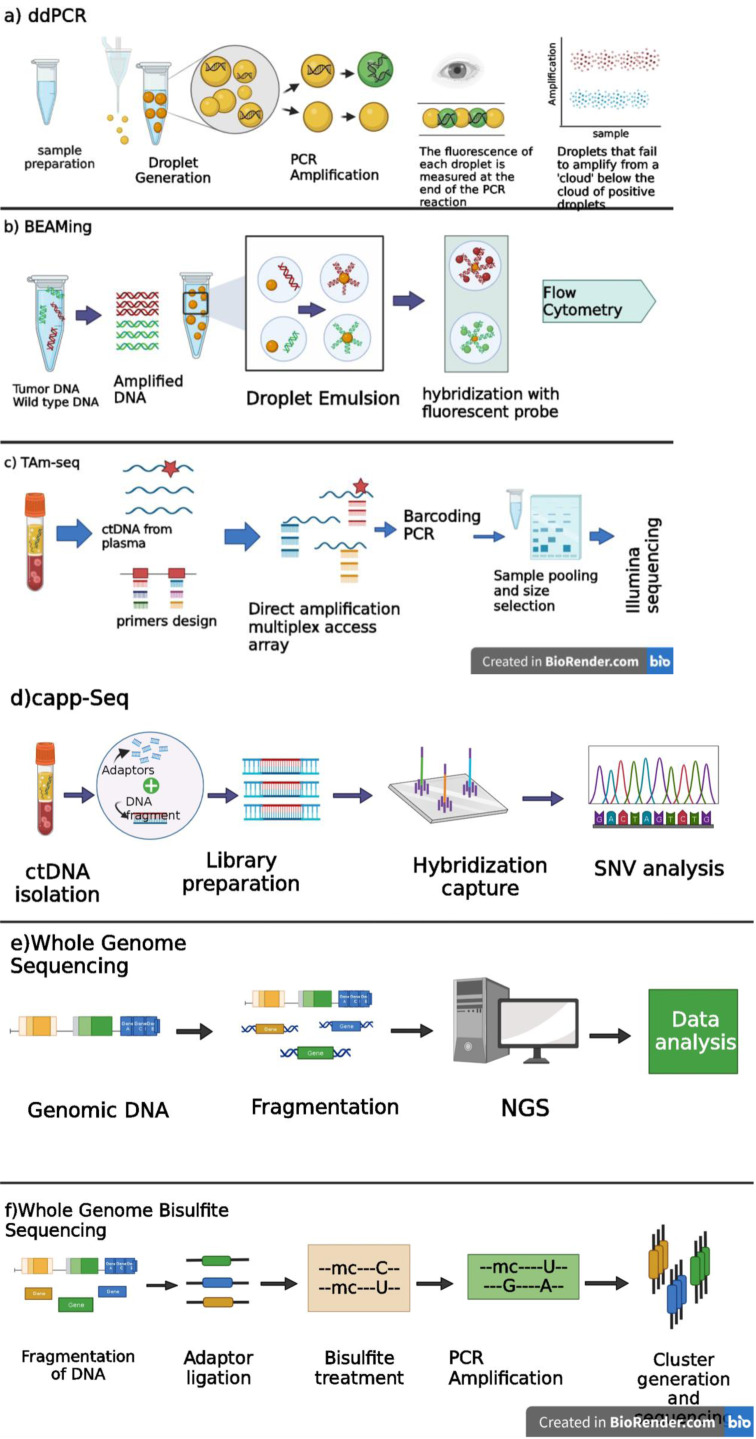
a) ddPCR workflow. The ddPCR methodology involves the partitioning of samples into numerous droplets undergoing amplification. Following the completion of PCR cycling, the fluorescence emanating from individual droplets is assessed. The droplets are categorized into positive or negative groups based on their fluorescence amplitude levels (y-axis) as the resulting output. b) BEAMing workflow. Multiplex PCR pre-amplification is used to amplify specific regions of interest in a DNA sample containing both wild-type (green) and mutant (red) copies. Emulsion PCR is then performed using magnetic beads, enabling millions of PCR reactions in micro-droplets. The beads are recovered, and their PCR products are denatured, leaving one DNA strand per bead. Fluorescence-labeled probes are attached to the DNA, distinguishing beads from PCR products. After washing, the beads are analyzed using flow cytometry to detect the presence of wild-type and mutant sequences. c) TAm-seq workflow. This technique utilizes the primers designed and multiplexed for direct amplification in ctDNA extracted from plasma samples. The amplification process is conducted using Fluidigm Access Array™ technology. The amplified DNA fragments obtained through PCR are subjected to a secondary PCR reaction for barcoding. The resulting samples are combined and experience a process of size selection in preparation for sequencing on an Illumina Hi-Seq 4000 platform. d) CAPP-seq workflow. In library prep, small synthetic DNA sequences called adapters are bonded to DNA fragments that will later be collected and sequenced. By using a multiphase bioinformatics technique, the adaptor is chosen. A probe-based hybridization capture is carried out on tumor and normal DNA using the Adaptor to find patient-specific mutations. The previously identified mutations are then quantified using the hybridization capture method on the ctDNA. e) WGS: whole genome sequencing workflow. The ctDNA is extracted from a sample and fragmented. Adapters are added to create a DNA library, loaded onto a sequencing platform, generating millions of short DNA reads. These reads are aligned to a reference genome or assembled *de novo*. Variations in the DNA sequence are identified through variant calling, followed by bioinformatics analysis. The genomic information is then interpreted based on the specific research or clinical context. f) Whole Genome Bisulfite Sequencing (WGBS) workflow. DNA is isolated and fragmented at the first step; then bisulfite is treated to differentiate methylated and un-methylated cytosines. The transformed DNA is sequenced and mapped to a reference genome to determine cytosine methylation. WGBS shows DNA methylation patterns and helps explain epigenetic regulation in numerous biological processes and illnesses. Created with BioRender.com

ddPCR technique presents a higher capacity for processing samples than qPCR while maintaining a similar cost per sample. However, the preliminary investment required for the ddPCR instrument remains too expensive (Teh et al., 2008[152]). Moreover, it is imperative to have proficient experts who can conduct the task and assess the outcomes. A significant impairment relates to the absence of easily accessible microfluidic technologies. In developing countries, where individuals are more vulnerable to viral infections and cancers, the accessibility of various reagents and equipment is often limited (Kojabad et al., 2021[88]). The potential emergence of novel methodologies may pose specific challenges for ddPCR. As the process improves, some parts may be removed or changed. One of the primary restrictions of ddPCR is the identification of false-positive partitions in no template control (NTC) wells (Biron et al., 2016[13]; Long and Berkemeier, 2020[98]). The etiology of the false droplet observed in non-template control samples remains unknown; however, implementing uncomplicated strategies such as adjusting the fluorescence threshold via software and bioinformatics pipelines can effectively moderate any potential biases (Kojabad et al., 2021[88]) (Figure 3a(Fig. 3)).

### Beads, Emulsion, Amplification, Magnetics (BEAMing)

BEAMing is a digital PCR method based on beads, emulsions, amplification, and magnetics. In this method, water-in-oil emulsions are used for condition optimization. In the second step, some magnetic microbeads coated with a specific primer gene are connected to DNA. Then, using a DNA polymerase, the target DNA is amplified, and the gap between the DNA is completed by ligase. In the third step, the flow cytometry technique helps to diagnose the mutant and wild DNA (Müller et al., 2006[108]; Vidal et al., 2017[159]).

One of the distinctive characteristics of BEAMing is that every produced microscopic emulsion droplet comprises an isolated DNA molecule and a magnetic bead coated with a primer. After the conventional PCR amplification process, the bead is enveloped with numerous target DNA fragments to furnish a digital readout of copy numbers, which can be subsequently analyzed using flow cytometry. The BEAMing technique detects rare mutant DNA in a significant background of wild-type genes, with a ratio of 1 mutant to every 10,000 wild-type genes (Taniguchi et al., 2011[148]).

BEAMing has been utilized to identify various ctDNA mutations, such as PIK3CA, BRAF, EGFR, KRAS, NRAS, IDH1, and ESR1. This technique has exhibited a substantial agreement between ctDNA present in blood samples of patients and somatic mutations detected in tumor tissue. BEAMing is a digital PCR-based analysis platform that, similar to ddPCR, exhibits higher sensitivity than other assays for detecting target sequences. It is well-suited for monitoring disease progression based on absolute circulating tumor DNA (ctDNA) quantitation (Chen et al., 2020[23]).

Similar to ddPCR, the BEAMing methodology exhibits certain disadvantages. A critical limitation of this approach is its cost, which is relatively higher than that of PCR. Furthermore, the BEAMing technique requires specialized equipment and skilled personnel to perform the assay. The number of beads that can be analyzed in a single reaction is also a limitation of the BEAMing method. This restriction can be challenging when analyzing samples containing low concentrations of target DNA. Additionally, the number of mutations detected in a single reaction is a limitation of the method (Denis et al., 2017[35]) (Figure 3b(Fig. 3)).

### Targeted Amplicon Sequencing (TAm-Seq) 

TAm-Seq, which stands for Tagged-amplicon deep sequencing, is a method that combines highly efficient library preparation techniques and advanced statistical analysis algorithms. The current process has been modified to perform sequencing, identification, and measurement of hepatocellular mutations throughout a gene panel encompassing not only tumor hotspots but also complete coding regions of specific genes (Forshew et al., 2012[47]).

Utilizing a bioinformatic approach has provided a distinct benefit in facilitating the creation of gene panels with enhanced efficiency, increased sensitivity in detecting mutant alleles, and minimized the occurrence of false positives. 

This technique is used to detect rare mutations in ctDNA. Tam-seq contains two processes: i) by pre-amplification step, all primer sets are used together to capture the starting molecules present in the template, and ii) the regions of interest in the single-plex reaction are selectively amplificated (Forshew et al., 2012[47]).

Achieving an optimal balance between the analyzed size of the genomic region and the sensitivity and specificity of the ctDNA assay is crucial in its clinical utility. When expanding the size of the genomic area under consideration, more rigorous alterations for false positive results are required. Enrichment methods based on hybrid capture have facilitated the examination of targeted genomic regions, including whole exomes. 

The TAm-Seq technique is utilized for detecting low-frequency mutations in cell-free DNA. It is an amplicon-based approach carefully adjusted to enable adequate amplification from restricted amounts of fragmented plasma DNA. However, the limitation of this methodology is its applicability solely to identified mutations. It cannot be employed for infrequent or unknown mutations (Gale et al., 2018[50]) (Figure 3c(Fig. 3)).

### Cancer personalized profiling by deep sequencing (CAPP-Seq)

The CAPP-Seq technique was designed to identify small amounts of ctDNA by utilizing "selectors" comprising biotinylated DNA oligonucleotides corresponding to previously established areas of recurrent mutations. The process of hybridizing the "selectors" on the region of interest is subsequently implemented by deep sequencing, enabling the identification of various mutations such as single nucleotide variants, rearrangements, and copy number alterations through CAPP-Seq.

CAPP-Seq works based on NGS for the detection of ctDNA. This method has excellent sensitivity and specificity to detect all main classes of mutations, including single nucleotide variants, copy number alterations, rearrangements, and indels (Bratman et al., 2015[16]). The hybrid capture-based NGS method is the advantage of CAPP-seq (Kato et al., 2021[82]). This technique achieves the lowest background error rate and detection limit of any NGS-based method used for ctDNA analysis by incorporating optimized library construction and bioinformatics methods (Newman et al., 2014[110]).

The utilization of CAPP-Seq for quantifying ctDNA has the potential to address several limitations associated with imaging techniques in assessing disease burden. Studies have shown that alterations in ctDNA concentrations can accurately measure therapeutic efficacy in individuals with progressive cancers. The CAPP-Seq methodology has been developed to restrict sequencing expenses by targeting frequently mutated genomic regions. Currently, this approach's substances and sequencing costs are estimated to range between $ 200 and $ 400 per assay (Bratman et al., 2015[16]). It is expected that these costs will decline as next-generation sequencing technologies advance. However, it is essential to consider several limitations when assessing the potential effectiveness of ctDNA monitoring for disease burden. The release rate of ctDNA from primary, nodal, and distant metastatic sites remains unclear. There will probably be some variation due to differences in tumor cell biology and the ability to reach circulation (Bettegowda et al., 2014[12]). The influence of tumor histology on ctDNA release remains incompletely known. Also, a further investigation involving significantly larger groups of patients is required despite encouraging data indicating the potential superiority of ctDNA analysis in terms of sensitivity compared to medical imaging (Bettegowda et al., 2014[12]). Furthermore, the analysis of circulating tumor DNA in isolation is insufficient to determine the specific anatomical sites of tumor deposits throughout the body. The analysis of ctDNA is expected to complement conventional imaging techniques in the context of disease surveillance (Diehl et al., 2008[41]) (Figure 3d(Fig. 3)).

### Whole genome sequencing (WGS)

Whole-genome sequencing (WGS) and whole-exome sequencing (WES) examine ctDNA through non-specific screening approaches. These methods are designed to comprehensively analyze copy number aberrations (CNAs) or point mutations across the entire genome.

WGS is an expensive but robust method for detecting across broad areas of the genome (Subhash et al., 2022[143]). A thorough tumor genome analysis called WGS or WES aims to find uncommon mutations and support clinical treatment. However, WGS is used to identify malignant mutations and therapy-induced changes in the initial tumor and influence future tumor genome analysis. Similarly, high-yield WES detect rare diseases and tumor cell mutations. It is only valid at a later stage of tumor detection (Li et al., 2021[95]). Next-generation sequencing (NGS) provides various benefits, including reduced sequencing time and cost and detailed genome analysis through the simultaneous sequencing of millions of DNA molecules (Gauri and Ahmad, 2020[54]).

Using untargeted strategies offers certain benefits, such as the capacity to detect new alterations that arise during tumor therapy and the absence of a prerequisite for prior knowledge regarding the genome of the primary tumor (Gilissen et al., 2014[58]). Nevertheless, a drawback of this approach is that it requires greater concentrations of ctDNA to ensure dependable reconstruction of tumor-specific alterations across the entire genome. Also, these methods are distinguished by their high prices and the requirement of the availability of highly developed technical resources as well as proficiency in bioinformatics. In addition, non-specific methodologies exhibit a reduced ability to detect the target analyte, with a sensitivity range of 5 % to 10 % (Glenn, 2011[59]).

The utilization of WGS and WES technologies results in incidental findings, which can be classified into two categories: (1) the accidental identification of germ-line mutations, resulting in ethical and legal challenges; and (2) the identification of wild-type variants with ambiguous or unknown clinical implications, including the identification of random "passenger" mutations. Nevertheless, this condition can be considered advantageous in cases where the examination aims to demonstrate the heterogeneity of cancer and the progression of clones, thus facilitating the creation of a phylogenetic tree of clonal expansion (Denis et al., 2017[35]).

These techniques are recommended for specialized genetic platforms and of significant scale. Despite the potential reduction in costs and complexity, implementing these approaches would only be possible in a limited number of clinical laboratories (Figure 3e(Fig. 3)).

### Whole-Genome Bisulfite Sequencing (WGBS-Seq)

The stable epigenetic modification of DNA methylation at the fifth position in cytosine (5mC) is a prevalent feature in various living organisms, ranging from bacteria to higher eukaryotes (Smith and Meissner, 2013[138]). In mammals, the genome is typically characterized by high levels of methylation. Specifically, in human embryonic stem cells (hESCs), up to 80 % of CpGs undergo DNA methylation, while the remaining unmethylated CpG residues are concentrated in CpG islands (CGI) situated at gene promoters (Chen et al., 2011[22]). Since it started in 1992, bisulfite (BS) sequencing of DNA has emerged as the standard approach for studying DNA methylation. The treatment of DNA with bisulfite (BS) results in converting unmodified cytosines to uracil while preserving the 5-methylcytosine (5mC) in its original form (Frommer et al., 1992[48]). This process enables mapping DNA methylation patterns at a single base resolution, following PCR and sequencing. In recent times, the utilization of next-generation sequencing (NGS) combined with BS treatment has resulted in the production of reduced representation (RRBS) or whole genome (WGBS) data. This data provides insights into the worldwide genomic distribution of 5mC (Olova et al., 2018[116]).

Because of its excellent accuracy and single cytosine measurement resolution, WGBS-seq has become the gold standard for DNA methylation evaluation. However, scaling up this technology for significant population research is prohibitively expensive and creates nontrivial bioinformatic problems (Wardenaar et al., 2013[162]). WGBS-Seq has significantly contributed to finding partly methylated regions in cancer cells. 

A limitation of WGBS-seq includes the decreased read depth resulting from alignment issues in specific genomic regions. In informatics, a higher cut-off for CpG density is frequently employed to reduce interference and enhance the statistical strength of the analysis (Chatterjee et al., 2017[21]). MeDIP-Seq, an early DNA methylation analysis technique, utilizes immunoprecipitation with a methylated cytosine antibody followed by next-generation sequencing (Ben Maamar et al., 2021[11]). Prior research has indicated that this approach is biased toward CpG regions with lower density in contrast to a method that employs methylated DNA binding proteins (MBP), which is inclined towards CpG regions with higher density. MeDIP-Seq exhibits limitations, such as lower throughput capacity and greater technical complexity than chromosomal bisulfite-based protocols (Nair et al., 2011[109]). Because of the bias toward higher density CpG, the percentage of the genome studied (e.g., 40 %) is lower than the MeDIP methodology, which is further lowered due to alignment difficulties (Beck et al., 2022[10]) (Figure 3f(Fig. 3)).

### ctDNA application in HCC diagnosis and therapy follow-up

Analyzing the mutational landscape of ctDNA from solid tumors may enable the detection of tumor-associated somatic mutations. One application that might be envisioned as having clinical value for hepatocellular carcinoma is identifying the malignant/metastatic pathways transformation based on the fundamental risk factor and genetic predisposition (HCC). Among these applications are the detection of genomic abnormalities, mutational analysis, prognostication, identification of oncogenic pathways, prediction and monitoring of treatment outcomes, changes in drug resistance, and the discovery of mechanisms underlying malignant/metastatic mutation (Kaseb et al., 2019[81]). Evidence suggested that ctDNA might be used as a liquid biopsy for various purposes, such as early diagnosis, predicting disease progression, and providing individualized care (Diaz Jr and Bardelli, 2014[40]). Additionally, ctDNA allows for the early detection of metastases during tumor growth. The likelihood of the disease recurring is also predicted by ctDNA change and the growing amount of ctDNA in the early stages (Deryugina and Kiosses, 2017[37]; Yong, 2014[174]). It has unequivocally been demonstrated that ctDNA detection correlates with the cancer stage. Comparing stage I to stages II-IV, the detection rate is 100 % (Bratman et al., 2015[16]; Yang et al., 2018[169]).

## Limitations

Despite the considerable research on ctDNA as a potentially effective "real-time" method for tumor characterization, medical practitioners still face several challenges that must be overcome before it can be widely employed (Yi et al., 2017[172]).

Sensitivity and specificity are two issues that significantly affect ctDNA analysis. Due to the incredibly low blood ctDNA, this molecule is not usually visible in peripheral blood. Additionally, smaller fragments produced from tumors cannot be well detected by the present digital PCR method, which prefers quantifying comparatively longer fragments, i.e., >120 bp (Norton et al., 2013[113]). Another issue is that employing fluorescent in situ hybridization (FISH) and immunocytochemistry (ICC), in situ, and morphological assessments are impossible. The variability and low ctDNA levels in plasma lead to various detection thresholds. Negative ctDNA results could also be caused by low copy number detection rather than a lack of ctDNA. In patients with resected early-stage cancer, whose plasma ctDNA levels are low, the inadequate sensitivity of the ctDNA assay is a severe problem. Due to the influence of biological factors such as mucinous histology, low DNA-shedding tumors, and concealed micro-metastases, false negative results are unavoidable. NGS panels may also improve assay sensitivity with a broad range of genomic or epigenetic modifications, more considerable sample volume, monitoring multiple mutations, serial testing, and fragment size analysis (Peng et al., 2021[121]).

Despite multiple translational research initiatives and a few clinical trials, doctors do not now routinely use the detection of ctDNA in patients with cancer. As a result, reliable commercialized biological tests have not yet been produced using various detection and analysis techniques. Furthermore, it appears premature to authorize these tests in a biological laboratory. The control of the pre-analytical phase, such as control of the various factors existing from the blood sample to the analysis of the ctDNA, is unquestionably one of the critical restrictions of this sort of detection (Devonshire et al., 2014[38]; Gao et al., 2022[52]; Pinzani et al., 2010[124]).

## Points of Dispute

The cell-free blood fraction contains circulating DNA fragments with tumor-specific sequence changes (circulating tumor DNA, ctDNA), which comprise a variable and a typically minor portion of the total circulating cell-free DNA (cfDNA). As a safe, minimally invasive alternative to tissue, tumor genotyping utilizing ctDNA may have advantages, especially in metastatic cancers. Prior research has shown a strong correlation between somatic mutations in tumor tissue and those in the ctDNA of patients with advanced malignancies. Additionally, ctDNA has been shown to help predict how well chemotherapy will work. Circulating tumor DNA may be a sign of post-resection residual disease and can be used to determine which patients are at risk for a relapse. After surgery and before adjuvant therapy, ctDNA testing should be done for the best therapeutic support decision-making (often 6 to 8 weeks following surgery) (Diaz Jr and Bardelli, 2014[40]). However, HCC diagnosis based on ctDNA has certain advantages and disadvantages. For instance, the interpretation of the clinical observation in cfDNA assays will be impacted by the contributions of cfDNA from apoptosis, necrosis, autophagic cell death, and active release at various time points during disease progression, treatment response, and resistance manifestation (Ye et al., 2019[171]). Other difficulties include the inability to identify ctDNA mutations in early-stage cancers with lower tumor burden (Ignatiadis et al., 2021[74]), complex variants of gene fusions (Heidrich et al., 2021[65]), the absence of HCC-specific mutation hotspots for detection by insufficiently broad NGS panels, and the lack of correspondingly effective treatments for druggable targets. The lack of uniformity also hampers its use in standard clinical practice in liquid biopsy methods (e.g., blood collection volume, tube types, sample storage, and shipping logistics) due to differing ctDNA profiling practices in various healthcare facilities (Zhang et al., 2021[176]).

## Future Studies

Personalized indicators based on the unique mutational profiles of resected tumors may be provided in future research examining postoperative levels of ctDNA, producing extremely high specificity. The ability to detect tiny amounts of ctDNA created from micro-metastatic deposits undetected by imaging or other diagnostic modalities will determine sensitivity. Applications of such an approach may affect a variety of cancers being managed to cure them (Diaz Jr and Bardelli, 2014[40]). The main difficulties limiting the use of ctDNA-based liquid biopsy for early detection are the low signals released by early-stage tumors, which directly impact the sensitivity of sequencing approaches (Christensen et al., 2019[27]; Razavi et al., 2019[127]; Reinert et al., 2019[128]). Additionally, the size selection of ctDNA fragments and the single-strand DNA library for NGS are effective remedies. Another concern is false-positive results when multiple alterations are sequenced using NGS (Newman et al., 2014[110]). The potential of errors being introduced during library preparation and subsequent sequencing stages is decreased by error-suppression approaches, such as using molecular barcodes and pipelines for bioinformatic interpretation of the data. It is envisaged that essential pre-analytical procedures, such as the collection, conservation, centrifugation, and extraction protocols for cfDNA, would be improved in addition to analytical methods (Gao et al., 2022[52]). The development of machine learning has increased the use of more signals and the integration of numerous features to enhance the discovery and detection of cancer-specific signals (Eraslan et al., 2019[44]). To facilitate the building of a diagnostic classifier based on the selected parameters, machine learning techniques include unchangeable approaches like logistic regression and advanced artificial neural networks using multi-dimensional data. Many liquid biopsy tests have already been improved through these approaches, making it simpler to include them in future clinical workflows (Sharif et al., 2020[136]).

## Conclusion

The current state of knowledge on ctDNA and HCC diagnosis methods is summarized in this review. We also go through the gene mutations that can aid in detecting ctDNA in HCC patients. Physicians can diagnose patients and administer efficient treatments by being aware of common ctDNA mutations. Additionally, identifying ctDNA in patients' blood using an efficient and effective diagnostic technique lessens the expense and suffering of ineffective treatments. Also, it aids in understanding how and how quickly the tumor advances.

## Declaration

### Acknowledgments

Authors would acknowledge Hematology and Oncology Research Center for their great help.

### Data availability statement

The data that support the findings of this study are available from the corresponding author upon reasonable request.

### Authors' contributions

NG wrote the manuscript, MSSZ edited the manuscript, JM gathered the required studies, AM supervised the project and guided the process, KA designed the figures and supervised the project. All authors read and approved the final manuscript.

### Conflict of interest

The authors declare no conflict of interest. 

## Figures and Tables

**Table 1:**
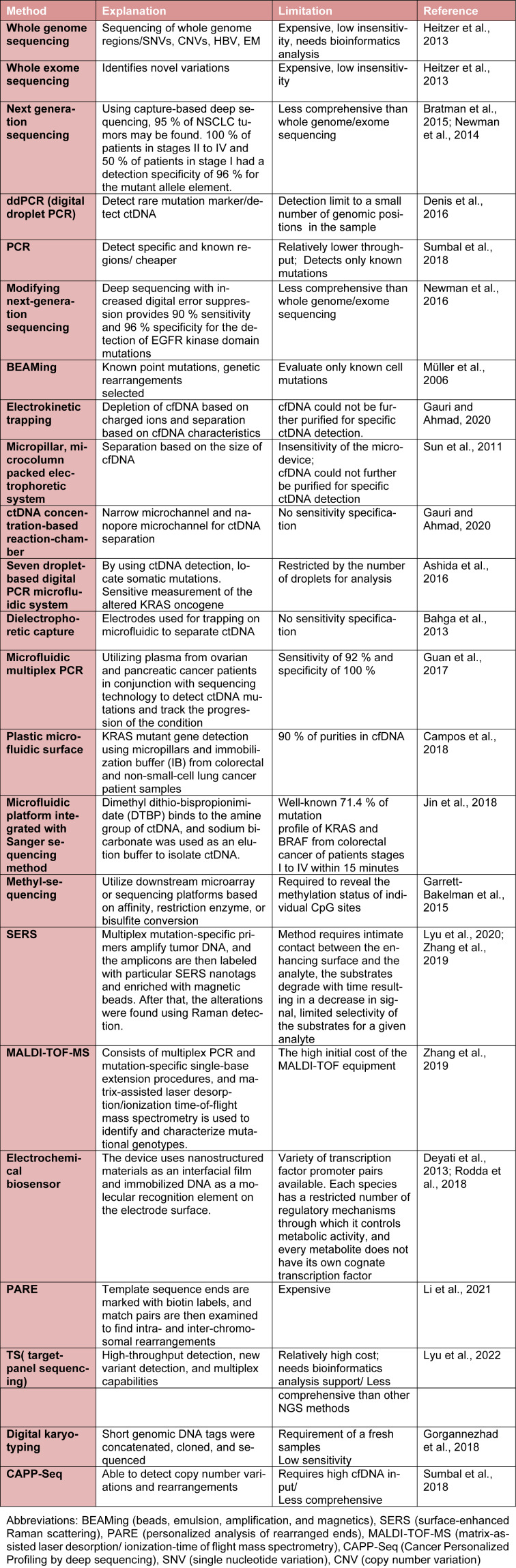
Methods of ctDNA diagnosis
